# Standardized massage interventions as protocols for the induction of psychophysiological relaxation in the laboratory: a block randomized, controlled trial

**DOI:** 10.1038/s41598-020-71173-w

**Published:** 2020-09-08

**Authors:** Maria Meier, Eva Unternaehrer, Stephanie J. Dimitroff, Annika B. E. Benz, Ulrike U. Bentele, Sabine M. Schorpp, Maya Wenzel, Jens C. Pruessner

**Affiliations:** 1 Department of Psychology, Division of Clinical Neuropsychology, University of Constance, Constance, Germany; 2 Child- and Adolescent Research Department, Psychiatric University Hospitals Basel (UPK), University of Basel, Basel, Switzerland; 3 Centre for the Advanced Study of Collective Behaviour, University of Constance, Constance, Germany

**Keywords:** Neuroscience, Psychology, Biomarkers, Cardiology

## Abstract

Health and disease are strongly linked to psychophysiological states. While stress research strongly benefits from standardized stressors, no established protocol focuses on the induction of psychophysiological relaxation. To maintain health, functioning regenerative systems are however likely as important as functioning stress systems. Thus, the identification of validated relaxation paradigms is needed. Here, we investigated whether standardized massages are capable of reliably inducing physiological and psychological states of relaxation. Relaxation was indicated by changes in high frequency heart rate variability (HF-HRV), a vagally-mediated heart rate variability component, and repeated ratings of subjective relaxation, and stress levels. Sixty healthy women were randomly assigned to a vagus nerve massage (*n* = 19), a soft shoulder massage (*n* = 22), or a resting control group (*n* = 19). During the intervention, HF-HRV and subjective relaxation increased, while subjective stress decreased significantly in all groups. Both massage interventions elicited significantly higher HF-HRV compared to the control group. Accordingly, both massage protocols increased psychophysiological relaxation, and may serve as useful tools in future research. However, future work will have to determine which of several protocols might be used as a gold standard to induce a psychophysiological state of relaxation in the laboratory.

## Introduction

Standardized protocols (SPs) that elicit specific psychophysiological states in the laboratory offer many advantages. For example, they might prove helpful in identifying the dysregulation of a specific system. Typically, SPs allow for (a) a reliable and valid induction of psychophysiological states in a controlled setting, (b) the study of their underlying mechanisms, (c) the study of their emotional, cognitive and behavioral consequences^1^, (d) and the comparability of results between studies and across laboratories, while ideally being time- and cost-saving. As an example, stress researchers use a number of well-established SPs (e.g. combined dexamethasone/CRH test^2^, or Trier Social Stress Test^3^) to better understand the stress response and its role in health and disease. Overall, SPs have moved the field considerably ahead^1^. So far, a lot of available SPs have focused on *psychophysiological* *stress*. However, the ability to activate regenerative systems during times of recovery is likely as important to maintain health as the activation of stress systems during times of threat. As such, an adequate relaxation response is key. However, there is yet a lack of SPs that elicit *psychophysiological* *relaxation*.

Ideally, SPs for relaxation should trigger both, a physiological relaxation response, mainly mediated by the parasympathetic system (PNS), and a psychological relaxation response, demonstrable in changes in subjective relaxation, or stress ratings. These criteria are barely fulfilled by former approaches: Some have focused on relaxation-inducing, cognitive-behavioral programs (e.g. progressive muscle relaxation)^4^, yet, previous experience might alter the effectiveness of these interventions, and the overall duration usually exceeds 15 min. Others have used solely physiological stimuli triggering the PNS (e.g. cold facial immersion^5^). Since cold water exposure has extensively been used as a pain-, and stress-inducing stimulus^6^, its use in the context of psychological relaxation is questionable. Along that line, direct or transdermal electrical stimulation of the vagal nerve^7^ increases PNS activity, but whether these rather cost-intensive approaches also stimulate psychological relaxation is not clear. As vagal sensory neurons innervate parts of the head/neck area, and respond to stretch, pressure, and temperature^8^, tactile stimulation in this region possibly increases vagal activity non-invasively and non-aversively. In fact, neck massages induce a shift from SNS to PNS dominance in animals^9,10^, and increase PNS activity in human populations^11–13^. Specifically, moderate, but not light pressure massage increases PNS activity^13–15^, possibly by direct stimulation of the vagal nerve. In addition, massages that do not directly stimulate the vagus nerve (e.g. back or hand massages) are still psychologically relaxing^16,17^. Physiologically, these massages may increase PNS activity via the modulation of breathing patterns^16^: slow deep breathing might induce physiological relaxation by stimulating pulmonary afferent nerves that influence the autonomic nervous system via brain stem regions^18^. Combined with the social, and wellbeing aspects of massages^19^, both kinds of massages could be suitable to induce psychophysiological relaxation in the laboratory.

Our primary aim was therefore to investigate whether two standardized massage protocols induce psychophysiological relaxation through a combination of psychological and physiological factors. While one protocol emphasized the physiological component and aimed at maximally activating the PNS by putting moderate pressure on the head/neck area (vagus nerve massage, VNM), the second one emphasized the psychological and wellbeing component, and lightly stimulated the back/shoulder area (soft shoulder massage, SSM). The effects on PNS activity, as indicated by changes in high frequency heart rate variability (HF-HRV), and subjective relaxation and stress levels were compared to a resting control group (RCG). Overall, we expected lower subjective stress, and higher subjective, and physiological relaxation levels in VNM and SSM, compared to RCG. Based on previous findings^11,14^, we also hypothesized that the VNM leads to a higher physiological relaxation response compared to the SSM, indicating a dose–response effect of pressure receptor activation on vagal tone.

## Results

The variables age, heart rate baseline, STQ, sexual orientation, and session start were equally distributed across the groups, and hence no confounds were entered in subsequent analyses (see Table 1).

**Table 1:** Descriptive baseline statistics of the experimental conditions.

Variable	Vagus nerve massage (*n* = 19)	Soft shoulder massage (*n* = 22)	Resting control group (*n* = 19)	*p*
Age (years)	23.44 ± 3.37	22.46 ± 3.94	22.474 ± 2.89	0.607
Heart rate baseline^a^	72.56 ± 11.97	79.97 ± 12.34	76.676 ± 9.04	0.120
STQ^b^ (sum score)	32.95 ± 11.12	28.36 ± 10.22	33.263 ± 8.94	0.226
Sexual orientation [(predominantly) heterosexual/other]^c,d^	16/2	21/1	17/2	0.704
Session start (morning/afternoon)^c^	6/13	7/15	3/16	0.431

If not otherwise specified, a one-way Analysis of Variance by experimental condition was calculated to test whether experimental groups differed in respect to the listed variables. In these cases, data is expressed as *mean* ± *SD.*

^a^Heart rate baseline: Mean of the first and second part of the heart rate baseline measurement.

^b^STQ: Social Touch Questionnaire.

^c^Pearson’s Chi-squared test was calculated to test whether experimental groups differed in respect to the listed variable.

^d^Sexual orientation was measured on a 5-point scale (heterosexual, predominantly heterosexual, bisexual, predominantly homosexual, homosexual). *n* = 1 participant did not answer the question.

**Figure 1:**
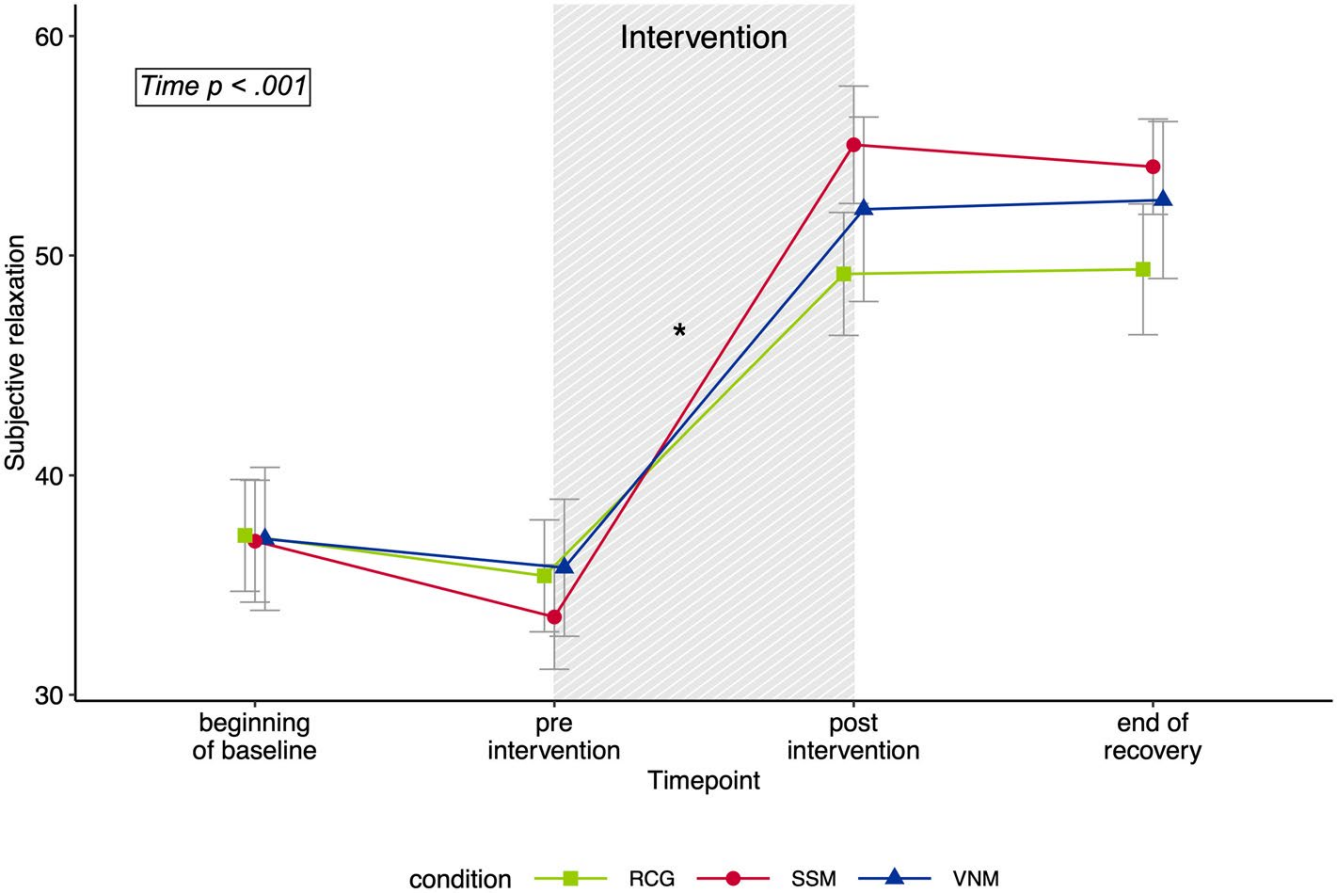
Changes in subjective relaxation ratings across the experiment in the three different experimental conditions vagus nerve massage (VNM; *n* = 19), soft shoulder massage (SSM; *n* = 22) and resting control group (RCG; *n* = 19). The intervention interval is shaded. Values are depicted as means ± SE. *Time* represents the within subject factor in the mixed Analysis of Variance. * indicates *p* < 0.001 in a post-hoc t-test comparing the timepoints pre intervention and post intervention across all experimental groups.

For the subjective stress outcome variable, the mixed ANOVA revealed neither a significant main effect of *Experimental Condition* (*F*_2,57_ = 0.09, *p* = 0.918, partial η^2^ = 0.003, BF_10_ = 0.258), nor a significant interaction between *Experimental Condition* and *Time* (*F*_2,57_ = 0.42, *p* = 0.657, partial η^2^ = 0.015, BF_10_ = 430,767.63), but a significant main effect of *Time* (*F*_1,57_ = 52.13, *p* < 0.001, partial η^2^ = 0.478, BF_10_ = 7.049E + 6). A post-hoc t-test indicated a significant reduction of subjective stress levels from pre (*mean* = 39.33, *SD* = 15.41) to post intervention (*mean* = 30.86, *SD* = 14.30) across all three experimental conditions (*t* (59) = 7.26, p_holm_ < 0.001, Cohen’s d = 0.938). Correspondingly, the mixed ANOVA investigating subjective relaxation indicated a significant main effect of *Time* (*F*_2.08,118.41_ = 60.54, *p* < 0.001, partial η^2^ = 0.515, BF_10_ = 2.662E + 24), but neither an effect of *Experimental Condition* (*F*_2,57_ = 0.22, *p* = 0.802, partial η^2^ = 0.008, BF_10_ = 0.116), nor a significant interaction between *Experimental Condition* and *Time* (*F*_4.16,118.41_ = 0.86, *p* = 0.479, partial η^2^ = 0.030, BF_10_ = 3.312E + 22). The changes of subjective relaxation ratings over time are shown in Fig. 1. Post-hoc t-tests indicated a significant increase in subjective relaxation levels from pre intervention (*mean* = 34.85, *SD* = 11.82) to post intervention (*mean* = 52.25, *SD* = 14.49) across all three experimental conditions (*t*(59) = 8.43, p_holm_ < 0.001, Cohen’s d = 1.09).

To test the hypothesis that receiving a massage (VNM and SSM condition) leads to greater increases in HF-HRV compared to rest (RCG), different statistical models with increasing complexity (see “Statistical analysis” section) were compared using an ANOVA. The successive incorporation of random intercepts (but not random slopes), the different trends of *Time* (linear, quadratic, cubic), and the first-order autoregressive covariance structure each led to significant increases in the model fit by means of the log-likelihood (see Supplementary Information, Table S1). Random slopes were therefore not included in the higher order models. After inclusion of the main effect *Touch Effect* (χ^2^(8) = 1.26, *p* = 0.262), the introduction of the interaction terms *Time* × *Touch Effect, Time*^2^ × *Touch Effect, and Time*^3^ × *Touch Effect* significantly improved the fit of the model (χ^2^(11) = 17.55, *p* < 0.001). While the condition model was 1.26 times more likely than the model with random intercept, fixed slope, and covariance structure, the interaction model was 18.81 times more likely than the model with random intercept, fixed slope, and covariance structure. The final model (Table 2) indicated that there was a significant difference in the HF-HRV trajectories over time for participants receiving a massage (VNM and SSM) compared to the RCG.

**Table 2:** Model parameters of the final model contrasting the massage groups against the resting control group.

	Standardized coefficient (*beta*) ± *SE*	*t* (*df*)	*p*
Intercept	− 0.18 ± 0.18	− 0.99 (349)	0.323
*Time*	2.56 ± 1.16	2.21 (349)	0.028
*Time*^2^	− 3.28 ± 0.97	− 3.40 (349)	< 0.001
*Time*^3^	− 1.21 ± 0.84	− 1.43 (349)	0.153
*Touch Effect*	0.27 ± 0.84	1.20 (58)	0.237
*Time *× *Touch Effect*	3.13 ± 1.40	2.23 (349)	0.026
*Time*^2^ × *Touch Effect*	− 1.47 ± 1.17	− 1.26 (349)	0.209
*Time*^3^ × *Touch Effect*	− 3.86 ± 1.02	− 3.77 (349)	< 0.001

*Time* represents the linear effect of time. *Time*^2^ represents the quadratic effect of time. *Time*^3^ represents the cubic effect of time. *Touch Effect* was entered as a dummy variable (resting control group = 0, soft shoulder massage = 1, vagus nerve massage = 1). *Time* × *Touch Effect* represents the interaction between the linear effect of time and *Touch Effect*. *Time*^2^ × *Touch Effect* represents the interaction between the quadratic effect of time and *Touch Effect*. *Time*^3^ × *Touch Effect* represents the interaction between the cubic effect of time and *Touch Effect.*

To test the hypothesis that the VNM leads to a greater increase in HF-HRV compared to the SSM, eight different statistical models with increasing complexity (described in the “Statistical analysis” section) were compared using an ANOVA. Like in the previous model, successive incorporation of random intercepts (but not random slopes), the different trends of *Time* (linear, quadratic, cubic), and the first-order autoregressive covariance structure each led to significant increases in the model fit by means of the log-likelihood (see Supplementary Information, Table S2). Random slopes were therefore not included in the higher order models. After inclusion of the main effect *Vagus Effect* (χ^2^(8) = 0.51, *p* = 0.474), the introduction of the interaction terms *Time* × *Vagus Effect, Time*^2^ × *Vagus Effect, and Time*^3^ × *Vagus Effect* did not significantly improve the fit of the model (χ^2^(11) = 0.81, *p* = 0.846). The condition model was 0.51 times more likely than the model with random intercept, fixed slope, and covariance structure. The interaction model was 1.33 times more likely than the model with random intercept, fixed slope, and covariance structure. The final model (see Table 3) indicated that, although HF-HRV changed significantly over time independent of the two massage groups, there was no significant difference in the HF-HRV trajectories over time for participants receiving the VNM compared to the SSM.

**Table 3:** Model parameters of the final model contrasting the vagus nerve massage group against the soft shoulder massage group.

	Standardized coefficient (*beta*) ± *SE*	*t* (*df*)	*p*
Intercept	0.01 ± 0.16	0.04 (236)	0.966
*Time*	5.27 ± 0.94	5.61 (236)	< 0.001
*Time*^2^	− 3.96 ± 0.78	− 5.05 (236)	< 0.001
*Time*^3^	− 4.39 ± 0.69	− 6.37 (236)	< 0.001
*Vagus Effect*	0.17 ± 0.24	0.70 (39)	0.488
*Time* × *Vagus Effect*	− 1.17 ± 1.39	− 0.84 (236)	0.399
*Time*^2^ × *Vagus Effect*	0.12 ± 1.17	0.10 (236)	0.918
*Time*^3^ × *Vagus Effect*	0.44 ± 1.02	0.43 (236)	0.665

*Time* represents the linear effect of time. *Time*^2^ represents the quadratic effect of time. *Time*^3^ represents the cubic effect of time. *Vagus Effect* was entered as a dummy variable (soft shoulder massage = 0, vagus nerve massage = 1; resting control group was not included). *Time*^3^ × *Vagus Effect* represents the interaction between the linear effect of time and *Vagus Effect*. *Time*^2^ × *Vagus Effect* represents the interaction between the quadratic effect of time and *Vagus Effect*. *Time*^3^ × *Vagus Effect* represents the interaction between the cubic effect of time and *Vagus Effect.*

**Figure 2:**
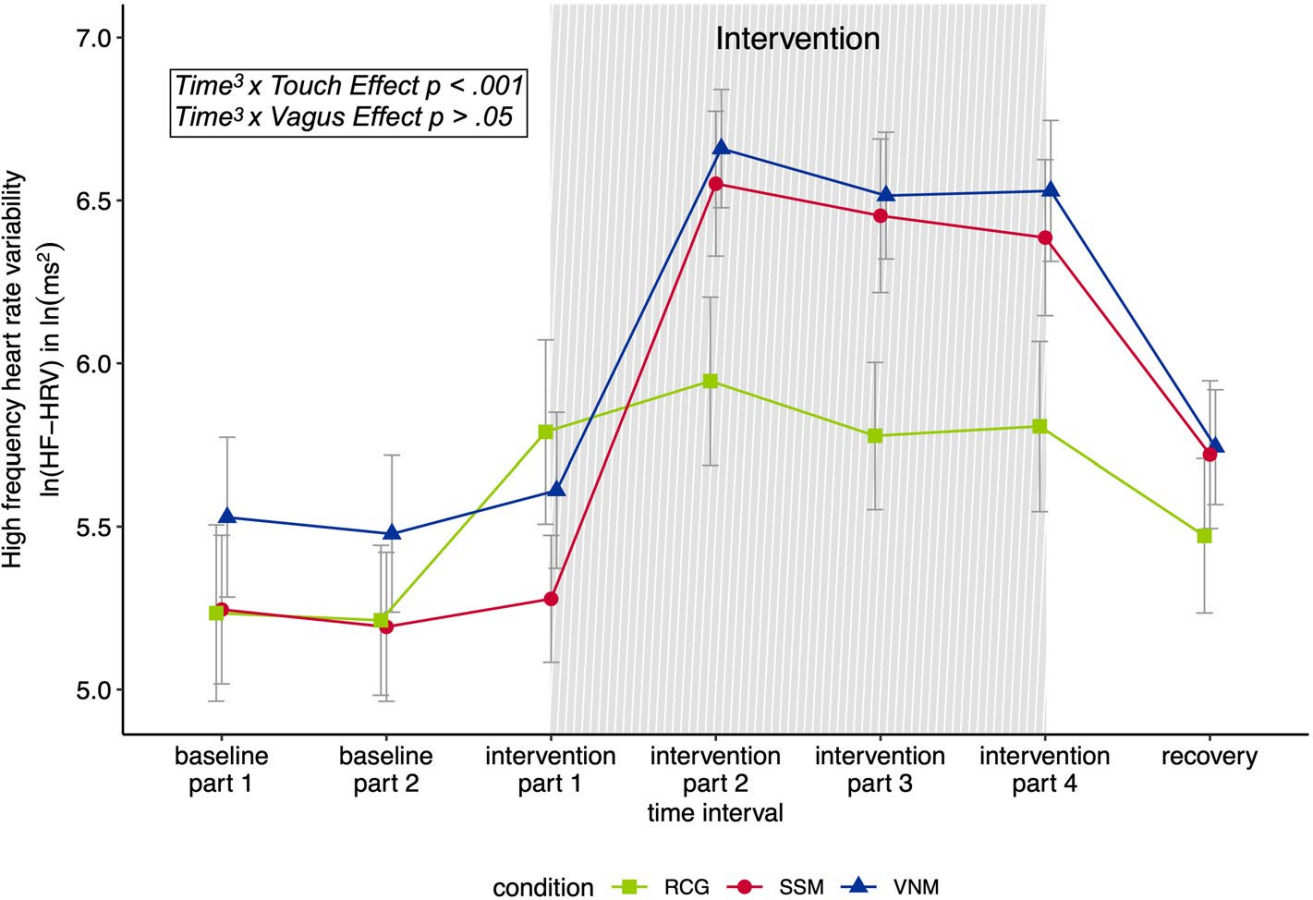
Changes in high frequency heart rate variability [ln(HF-HRV) in ln(ms^2^)] across the experiment in the three different experimental conditions vagus nerve massage (VNM; *n* = 19), soft shoulder massage (SSM; *n* = 22) and resting control group (RCG; *n* = 19). The intervention interval is shaded. Values are depicted as means ± SE. *Time*^3^ × *Touch Effect* represents the interaction between the cubic effect of time and *Touch Effect* (a dummy variable indicating resting control group = 0, soft shoulder massage = 1, vagus nerve massage = 1). *Time*^3^ × *Vagus Effect* represents the interaction between the cubic effect of time and *Vagus Effect* (a dummy variable indicating soft shoulder massage = 0, vagus nerve massage = 1).

The HF-HRV trajectories over *Time* for the different *Experimental Conditions* are depicted in Fig. 2.

As can be seen in Fig. 2, HF-HRV levels in the RCG seemed to change over the course of the experiment, with an increase during the time of the intervention. Since the main effects of the above described models can hardly be interpreted as soon as significant interactions are present, we exploratory calculated a model as described above with the data of the RCG only (without modelling a main effect of *Experimental Condition* and the interaction effect *Time* × *Experimental Condition*). The resulting model indeed confirmed significant main effects of *Time* (Standardised Coefficient (*beta*) = 4.70 ± 0.51, *t*(352) = 9.25, *p* < 0.001), *Time*^2^ (Standardised Coefficient (*beta*) = − 4.54 ± 0.51, *t*(352) = − 8.93, *p* < 0.001), and *Time*^3^ (Standardised Coefficient (*beta*) = − 3.85 ± 0.51, *t*(352) = − 7.60, *p* < 0.001), indicating a significant change of HF-HRV levels over the course of the experiment in the RCG. The model including a cubic effect of time was 174.80 times more likely than the model with random intercept only.

To complete the picture, and for comparative reasons, the same analyses were performed using RMSSD instead of HF-HRV. The detailed results can be obtained from the Supplementary Information. Overall, the results of these analyses reflect the results described above, without changes in significance.

## Discussion

At the core of the current study was the idea that—similar to the investigation of the stress system using standardized protocols (SPs)—triggering and recording a psychophysiological relaxation response using a standardized stimulus can be informative to investigate the potential dysregulation of the regenerative system in connection with health and disease. For the identification of a such a relaxation protocol, we investigated the effects of two standardized massages on changes in subjective stress and relaxation levels, and high frequency heart rate variability (HF-HRV) in a group of healthy, young females, compared to a resting control group. During the 10-min intervention, subjective stress levels decreased, while subjective relaxation levels and HF-HRV increased in all participants independent of the experimental condition. As hypothesized, both massage groups elicited a greater increase in HF-HRV compared to the resting control group. Contrasting the massage groups, the increase in HF-HRV was independent of the pressure applied to the skin, as both groups showed a comparable increase in HF-HRV during the intervention. Finally, a 10-min rest also led to a significant increase in HF-HRV, albeit less pronounced as compared to the massage interventions. Overall, the two standardized massage protocols achieved a mean increase in HF-HRV of 24.67% across participants, with 90.24% of them responding to the intervention with an increase in HF-HRV. In comparison, the mean increase in HF-HRV in the resting control group was 13.24%, with 84.21% of them responding to the intervention with an increase in HF-HRV. No participant indicated that the massage had negative short-term consequences (brief discomfort, nausea or dizziness).

Our results indicate that all interventions (both massages, and the 10-min rest) were capable of significantly increasing subjective relaxation, while decreasing subjective stress. This pinpoints at a high potential of short relaxation periods to alleviate states of psychological tension, and increase subjective relaxation.

Our results further revealed that there was no direct mapping between the psychological, and physiological relaxation response: While subjective markers did not differ between the experimental groups, the increase in HF-HRV was significantly higher in both massage groups compared to the control group. This discrepancy is in line with the often-reported lack of correspondence of the psychological and physiological responses to stress^20^. Potentially, this non-correspondence might also be evident in other psychophysiological states.

In line with previous work^11–13^, our data supported the hypothesis that the massage protocols lead to higher increases in HF-HRV compared to the resting control group. This further highlights that massages are potent modulators of psychophysiological responses^21–23^. In the vagus nerve massage group, these effects might be explained by tactile stimulation of vagal sensory neurons which innervate parts of the head and neck area^8^. However, the finding that both massage protocols were capable of increasing parasympathetic nervous system (PNS) activity to a similar degree partly contradicts previous findings showing that at least moderate, but not light pressure massage is necessary to increase parasympathetic activation^11,14^. It is not clear how much certain exercises that were used in both massages, for example the application of the warmed palms to the shoulder area, have contributed to the observed effects. The aforementioned exercise is likely leading participants’ attention towards their breath, so that a deeper and slower breathing pattern can be expected. Slow deep breathing has been shown to increase PNS activity^24,25^, which makes it difficult to tell at this point, whether the effects we have observed are solely due to changed breathing pattern, or due to other massage related mechanisms. In addition to that, there is evidence that slow and light stroking in hairy skin regions activates C-tactile cutaneous afferents. This subgroup of unmyelinated fibres transmits signals to areas that process positive emotional feelings and interoceptive awareness, such as the left anterior insular cortex^26^. As the standardized soft shoulder massage protocol included elements of effleurage massage techniques (circular, stroking movements with the palm of the hand to warm up the muscles), this stimulation could have triggered C-tactile cutaneous fibres that indirectly mediated a relaxation response^27,28^. Besides the effects of the massage itself, it is certainly conceivable that the positive social interaction, and the attention received from the experimenter in both massage groups contributed to the relaxing effect, for example by triggering the release of oxytocin^9^, or activating the default mode network^29^ via interpersonal touch. At this point however, these considerations are speculative and await further experimental validation. Taken together, it is unclear whether the enhanced physiological relaxation induced by the massages was solely due to the tactile stimulation, and which other factors might play a role here.

Finally, we want to highlight that the resting position that was taken up in the control group also induced a significant increase in psychophysiological relaxation, albeit less pronounced on the physiological level. This result emphasizes how deflectable the PNS can be in healthy participants: Even slight changes in sitting position and attention lead to significant changes in vagal modulation^30^. We assume that this resting position could be used when the research question focuses on effects of self-induced relaxation which are independent of the effect of tactile stimulation, and social interaction.

Besides the discussion of the results, a number of limitations of the reported study should not remain unmentioned here. Although we have screened for some health parameters that could potentially bias the effects of massages on PNS (re)activity, our exclusion criteria for this student sample were rather liberal. Potential confounders such as obesity (body mass index > 40), depressiveness, menstrual cycle phase, as well as the status of certain relevant clinical conditions such as autoimmune or endocrine disorders were neither assessed, nor used as exclusion criteria. These variables can influence the functioning of the autonomic nervous system^31^, and future studies should aim to address their potential effects. In addition, stricter control of sampling time, and meal intake before sampling could minimize potential effects related to circadian rhythm and digestion. Although the study was conducted in the same room throughout the data collection, no objective monitoring of room temperature and humidity was conducted. Both measures should be included in future studies to control potential effects and facilitate replication. Further, an objective measure of respiratory rate could inform about the effect of breathing pattern on HF-HRV^30^.

A more general limitation of employing massage techniques as SPs to induce a relaxation response has to do with potential effects of sex, gender and sexual orientation. Although we strived to induce a non-sexual, hedonic tactile experience in both massage procedures, we additionally controlled for the influence of sex and gender by inviting female participants, and employing female experimenters. However, we did not control for sexual orientation. With only a small subset of the sample (*n* = 5) reporting that they identified themselves as either bi- or (predominantly) homosexual, the small group sizes have prevented statistical conclusions. The possible effects of sex and sexual orientation may restrict the generalizability of the current results, and raise the importance to investigate on the generalizability of the examined protocol.

Another critical concern is that massages might overall represent an approach that poses challenges in standardization across laboratories. Although we can provide SPs (see Supplementary Information), the variation in interpretation by different experimenters might still lead to slightly differing procedures, and the effect of these differences are to be quantified in future research. To remedy this, it might be promising to test deep slow breathing as a standardized relaxation protocol. Although the physiological effect of such breathing exercises has been shown repeatedly (e.g.^24^), it remains an open question whether they also elicit a psychological relaxation response. Thus, building on previous results showing that controlled breathing exercises that are normalized by the spontaneous breathing rate of participants induce physiological relaxation^25^, testing the psychological effects of such protocols should become a focus of future research. However, we also must acknowledge that variations in stress protocols such as the Trier Social Stress Test (TSST) are existent^32^, and despite these differences, these protocols are used with great success. Taken together, the usefulness of the introduced massage protocols for the detection of dysregulations in the regenerative system and their link to health and disease has to be determined in the future.

In conclusion, our short, and easy-to-implement standardized massage protocols seem to provide a valid and reliable tool to investigate psychophysiological relaxation in healthy women. Since the external validity of this initial study is limited, follow up studies will have to test the generalizability of the relaxing effects of our SPs in more diverse samples, particularly in males. Building on that, the SPs allow the examination of cognitive and behavioral consequences of relaxation, while ensuring comparability between laboratories^1^. While stress research has long benefitted from SPs, our understanding of the psychophysiological relaxation response and its role in health and disease remains unclear at this point, but should be of high importance in the field of mental health. Hence, future researchers can determine which factors further explain between subject variability in psychophysiological relaxation. Here, it will be of special interest to investigate whether participants with dysregulated stress systems, as seen e.g. after a history of early life stress, also show dysregulated regenerative systems. Interestingly, there is evidence hinting at a modulation of the sensitivity of C tactile fibres by adult attachment style^33^, a concept that is related to early life stress. Future research will have to determine how, and which of the psychophysiological systems contribute to the development and progression of mental disease.

During the last decades, HRV has gained attention as a non-invasive and economic measures of PNS (re)activity. In light of these developments, our standardized relaxation protocols represent a valuable new research tool. Nonetheless, we want to further emphasize that this study is a first approach in the search of a standardized relaxation protocol. We could show that it is highly feasible to introduce such a protocol, however, future work will have to determine which of several SPs might be used as a gold standard to trigger a psychophysiological relaxation response in the laboratory.

## Methods

### Determination of sample size

Based on restriction of resources and feasibility considerations, we decided to assess a total of *N* = 60 participants, with 20 participants in each condition (vagus nerve massage, soft shoulder massage, resting control group), prior to conducting the study. To account for the potential exclusion of some participants, we assessed a total of *N* = 63 participants overall. After the exclusion of *n* = 3 (reasons see below), we achieved 80% power to detect effects sizes of at least *f* = 0.41 at a significance level of α = 0.05.

### Participants

**Figure 3:**
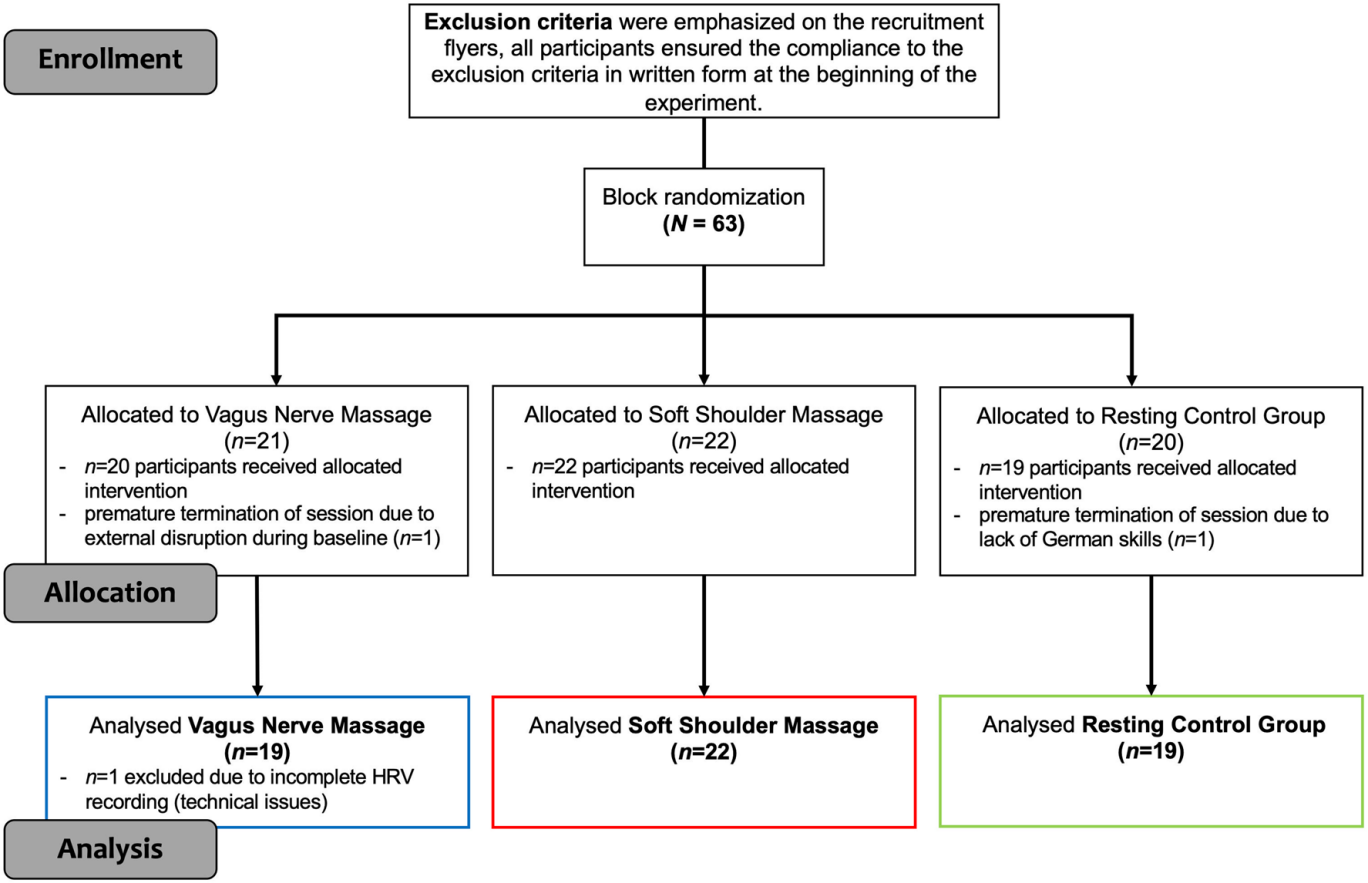
Flow diagram visualizing the sample of the study.

Sixty-three healthy, female participants were recruited at the University of Constance over a three-month period in the summer of 2018. To minimize the risk of negative side effects of the massage (brief discomfort, nausea or dizziness), and effects of potential modulators of the PNS, exclusion criteria involved past, or current orthopaedic problems (e.g. scoliosis, discopathy, spinal fracture), whiplash, thyroid or cardiac disease, intake of blood-thinning or anticoagulant medication, smoking and left-handedness. These criteria were emphasized on the recruitment flyers, and participants confirmed the compliance to these criteria in written form at the beginning of the experimental procedure. Interested male participants were not invited to control for possible sex effects in the massage giver/receiver interaction, since experimenters were all female. Eligible participants were assigned to either a vagus nerve massage (VNM), a soft shoulder massage (SSM), or a resting condition (resting control group, RCG) by the experimenters using block randomization (block size of three) in a parallel design to assure equivalent sample sizes among groups. Using a block size of three, it was determined prior to the recruitment of participants which experimental condition was tested on which day and time (no double-blind approach). Subsequently, participants signed up blindly to a session and were not informed about their allocation to a certain condition prior to the debriefing. Participants were asked to withhold from consuming any caffeine-containing beverages 2 h prior to testing. Three participants had to be excluded due to technical problems (*n* = 1), lack of German skills (*n* = 1), or disruption of the experimental procedure (*n* = 1). A flow diagram visualizing the sample is depicted in Fig. 3.

The final sample of 60 participants (*mean*_age_ = 22.76 years, *SD*_age_ = 3.43, *range*_age_ = [19, 36] years) consisted of students (*n* = 57; 95.00%), employed (*n* = 1; 1.67%), self-employed (*n* = 1; 1.67%), and unemployed healthy adults (*n* = 1; 1.67%). 61.67% (*n* = 37) were currently in a relationship (mean duration 2.47 years). The majority (*n* = 54 of *n* = 59) stated that they identified themselves as (predominantly) heterosexual (*n* = 1 participant did not answer that question).

### Procedure

**Figure 4:**
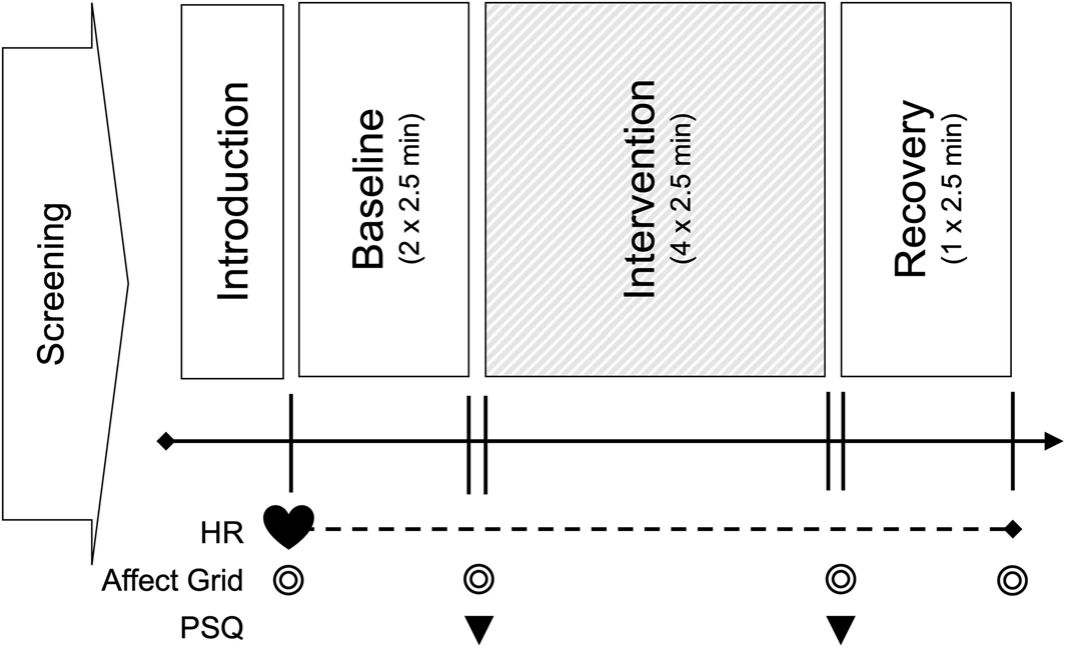
Overview of the study procedure. *HR* heart rate monitoring, *PSQ* Perceived Stress Questionnaire. During the intervention, participants were randomly assigned to either a vagus nerve massage, a soft shoulder massage, or a resting control group. For subsequent heart rate variability analysis, the periods of interest were split up into 2.5 min segments as depicted in the figure to allow for a higher temporal resolution of heart rate variability changes.

Experimental sessions were scheduled to start between 7:00 a.m. and 7:00 p.m. and lasted for approximately 50 min. The sampling took place in the same room throughout the entire study. The detailed study procedure is depicted in Fig. 4.

During an introduction, participants gave written informed consent, confirmed the compliance to the exclusion criteria in written form, and put on the chest strap for continuous heart rate (HR) monitoring. After that, participants sat down for an acclimatization period, filled in questionnaires and sat quietly for 5 min to obtain a physiological baseline. They were instructed to sit upright with both feet flat on the floor, and knees at a 90° angle. This was followed by the completion of a hand power test and a fine motor skills task (5 min) which are not analysed in the course of this work. Directly afterwards, a standardized 10-min intervention of either a VNM, a SSM, or a resting condition was carried out during which participants remained seated (RCG). After the completion of a creative task, and a post assessment of the hand power test, and the fine motor skills task (5 min) which are not analysed in the course of this work, participants completed the Social Touch Questionnaire at their own pace. This questionnaire period was considered as a recovery period.

Participants were asked to rate their current mood on the Affect Grid (described in detail below) at four time points during the study procedure and their perceived stress level on a modified version of the Perceived Stress Questionnaire at two time points. At the end, participants were thanked, debriefed and received compensation for participation.

### Experimental and control conditions

During the 10-min intervention, all participants were asked to sit comfortably, place their head on a massage cushion on the table, and close their eyes. Participants were informed that the massage would be interrupted immediately, as soon as they indicated any negative short-term consequences (brief discomfort, nausea or dizziness). The head was aligned as straight as possible to avoid a one-sided stimulation. Whenever questions occurred, conversations were restricted to a minimum; else the intervention phase was carried out in silence. The VNM group received a standardized head/neck massage that was applied with moderate pressure. The stimulation included stroking and twisting the trapezius muscle by gripping between the trapezius and sternocleidomastoid muscle, below which the main strand of the vagus nerve runs, to reach deeper regions. Further, the VNM involved stroking the muscles below the base of the skull from the spine to the back of the ears. In contrast, the SSM was applied by stroking and softly touching the neck and shoulder area. The RCG did not receive any physical contact. The SPs including detailed instructions for the VNM and the SSM can be obtained from the Supplementary Information.

### High frequency heart rate variability (HF-HRV)

RR intervals were collected continuously at a sampling rate of 1,000 Hz using a Bluetooth low energy Polar H7 heart rate sensor on a two-electrode chest strap (Polar, Finland) and the application Heart Rate Variability Logger for iOS^34^. RR intervals differing more than 20% from the previous interval were discarded by the application to correct for artefacts caused by noise or motion. HF-HRV of the events of interest (baseline, intervention and recovery) was calculated using the R package *RHRV*^35^.

HF-HRV reflects the temporal variation between successive heart beats that occurs in the respiratory frequency band^31,36,37^. It is one of the most commonly used vagally-mediated heart rate variability parameters^38^ which is generally accepted to validly reflect parasympathetic activity^31,36^. This has been shown in a number of foundational studies^39^ showing that scopolamine, a pharmacological block of the PNS, completely eliminates HF-HRV. In contrast, beta-adrenergic blockers, which block the sympathetic nervous system, have no influence on HF-HRV. Thus, a SP triggering a physiological relaxation response could be identified using HF-HRV as a biomarker of PNS activity.

For the extraction of HF-HRV, we employed an R script (in-house) that automatically derives 5 min of each subject’s baseline, 10 min of each subject’s intervention, and 2.5 min of each subject’s recovery phase. These segments were split up into 2.5-min intervals (with exception of the recovery phase segment) and the instantaneous heart rate signal was extracted for each segment. Artefacts were detected automatically by a filtering algorithm provided by *RHRV* that uses adaptive thresholding to reject beats whose RR value differ from preceding and subsequent beats more than a threshold value. The algorithm further removed values that are not within an acceptable physiological range^35^. In addition to that, RR intervals were visually screened, and ectopic beats were removed. After removing artefacts, the heart rate signal was interpolated at a sampling frequency of 4 Hz. In the subsequent frequency analysis, mean HF-HRV over the 2.5-min periods was calculated using 60 s segments with a shift of 30 s, and a fixed frequency bandwidth of 0.15–0.4 Hz. Since the duration of the recovery time (the time during which participants completed the Social Touch Questionnaire) was not standardized temporarily across participants, and to allow for a better comparability between subjects, we used a fixed window (2.5 min) for HF-HRV calculations. Since HF-HRV calculations are considered unreliable when the time period of RR assessment is too short, participants with a total recovery time interval shorter than 2.5 min (*n* = 3 of VNM, *n* = 1 of SSM, *n* = 1 of RCG) were assigned no HF-HRV value for this period. Finally, to account for the violation of the normality assumption, we calculated the natural logarithm of the resulting HF-HRV values and used them as an index of HF-HRV in all following statistical analyses. For readability reasons, HF-HRV is used to refer to ln(HF-HRV) throughout the manuscript.

Beside frequency domain heart rate variability components, time domain parameters such as the root mean square of successive differences (2) have been used frequently to describe sympatho-vagal balance of the autonomic nervous system^31^. In the course of the processing described above, RMSSD was calculated analogously to HF-HRV. Since we were particularly interested in changes in PNS activity, we will focus on the effects of HF-HRV in the following. RMSSD data can be obtained online at the Open Science Framework project associated with this work.

### Subjective levels of relaxation

Psychological relaxation was measured using the Affect Grid^40^, which assesses affective state along the two dimensions displeasure/pleasure, and arousal/sleepiness. The instrument has adequate reliability and validity^40,41^, and has been used in in different experimental settings, such as longitudinal ecological momentary assessment^42^, or experimental designs^43–45^. The score for each dimension ranges from 1 to 9. To analyse subjective levels of relaxation, we multiplied the pleasure and arousal scores to receive a single-item score, with higher scores indicating higher levels of subjective relaxation (range 1–81).

### Subjective levels of stress

Subjective stress levels were assessed using the revised, 20 item German version of the Perceived Stress Questionnaire (PSQ^46^), a widely used instrument in research on stress and well-being^47^. The questionnaire shows satisfactory reliability^46^, and validity^48^. We modified the temporal frame by asking participants to rate how strongly an item applied to them on a 4-point Likert scale (1 = does not apply at all, 4 = does entirely apply) in *current moment*. An overall sum score was calculated after inversing the items of the scale “joy”. The higher the score, the more stress subjects perceived within the situation (range 20–80).

### Social Touch Questionnaire

To assess the general attitude towards touch, participants completed the Social Touch Questionnaire (STQ^49^), a 20 item scale asking participants to rate how strongly an item applies to them on a 5-point Likert scale (0 = not at all, 4 = extremely). The instrument is frequently used in research on social touch and related areas (e.g.^50^) and is considered a reliable and valid instrument^49,51^. A sum score was calculated (range 0–80), with lower scores indicating a more positive attitude towards social touch.

### Statistical analysis

Statistical analyses were conducted in R version 3.5.3^52^ using RStudio version 1.1.463^53^ with the packages *ez*^54^, *nlme*^55^*,* and *car*^56^*,* and in Jeffreys’s Amazing Statistics Program^57^ version 0.11.1. Graphs were created using the R package *ggplot2*^58^. If required, normality assumption was checked using the Shapiro–Wilk Normality test and histograms of model residuals, and homogeneity of variance assumption was checked using Levene’s test. The level of significance was set at α = 0.05. Overall, *n* = 19 of the VNM, *n* = 22 of the SSM, and *n* = 19 of the RCG were included in the following analyses.

One-way ANOVAs (type II) with *Experimental Condition* (VNM, SSM, RCG) as independent variable were performed to ensure equal distribution of age, heart rate baseline (mean of the first and second part of the heart rate baseline measurement), and STQ across the three groups. Furthermore, Pearson’s Chi-squared tests were conducted to test whether the variables sexual orientation [(predominantly) heterosexual/other), and session start (morning/afternoon)] were equally distributed across the experimental groups. Variables that were not equally distributed across the groups were considered as potential confounds, and were controlled for in subsequent analyses.

First, we performed mixed analyses of variance (ANOVAs) with *Experimental Condition* (VNM, SSM, RCG) as between-subject factor, *Time* as within-subject factor and the outcomes *subjective stress* and *subjective relaxation* to assess whether the different interventions induced different changes in subjective stress, and relaxation levels. Greenhouse–Geisser corrections were applied whenever the sphericity assumption was violated according to the Maulchy’s test for Sphericity. Partial eta squared (partial η^2^) was used as indicator of effect size^59,60^. In addition, Bayes factors (BF_10_) were calculated in comparison to the null model, indicating the likelihood of the data given the alternative hypothesis is true divided by the likelihood of the data given the null hypothesis is true^61,62^.

Because of five missing values in HF-HRV during the recovery period and high inter-individual variations in HRV^31^, a growth curve approach was performed to assess changes in HF-HRV over time, estimating within-subject trajectories of HF-HRV over time on level 1, individual differences on level 2, and differences caused by experimental condition on level 3. To test the hypothesis that receiving a massage (VNM and SSM condition) leads to greater increases in HF-HRV compared to rest (RCG), experimental condition was entered as a dummy variable called *Touch Effect* (RCG = 0, SSM = 1, VNM = 1). In the baseline model, HF-HRV was predicted from the intercept. After that, random intercepts across individuals (random intercept model) were incorporated. Next, a linear fixed effect of *Time* (*Time* model) was included. Thereafter, random effects of *Time* over people (random slope model) were incorporated. Then, a quadratic trend of *Time* (*Time*^2^ model) and a cubic trend of *Time* (*Time*^3^ model) were added successively as orthogonal predictors, as HF-HRV was expected to show a curvilinear change over the course of the experiment. After that, a first-order autoregressive covariance structure was added to the model (random intercept, fixed slope model with covariance structure). After adding the condition effect *Touch Effect* (condition model), the interaction terms *Time* × *Touch Effect, Time*^2^ × *Touch Effect, and Time*^3^ × *Touch Effect* were entered into the model (interaction model). Resulting changes in overall model fit by means of the log-likelihood value were compared using an ANOVA and the final model was evaluated. As an estimate of effect size in nested models, we calculated the likelihood ratio of the condition model and the model with random intercept, fixed slope, and covariance structure, and the likelihood ratio of the interaction model and the model with random intercept, fixed slope, and covariance structure. As such, the likelihood ratio represents how much more likely the data is under the given model compared to the less complex model.

In order to calculate standardized regression coefficients (which are interpretable as effect sizes), we z-standardized the HF-HRV values before conducting the analyses.

To test whether the VNM leads to a higher HRV increase compared to the SSM, we excluded the RCG, and again entered experimental condition as a dummy variable called *Vagus Effect* (SSM = 0, VNM = 1) and subsequently ran the same analysis as described above.

### Ethics approval, written informed consent, and compensation

The study was approved by the Ethics Committee of the University of Constance, Germany, prior to its conductance (IRB statement 12/2017), and was carried out in accordance with the ethical standards of the Declaration of Helsinki. All participants gave written informed consent prior to participation and received financial compensation (€10). The study is officially registered at the German Clinical Trials Register (https://www.drks.de, trial number DRKS00021162, date of registration 27/03/2020).

## Data Availability

The dataset generated and analysed during the current study is available online at https://osf.io/4bwsj/ (Open Science Framework dataset 10.17605/OSF.IO/W6HTB). Supplementary information (e.g. a trial protocol associated with this work) is available online at https://osf.io/4bwsj/ (Open Science Framework project 10.17605/OSF.IO/4BWSJ). A preprint of this manuscript has been published on PsyArXiv (10.31234/osf.io/m85qc).
